# Racial Differences in Left Atrial Size: Results from the Coronary Artery Risk Development in Young Adults (CARDIA) Study

**DOI:** 10.1371/journal.pone.0151559

**Published:** 2016-03-17

**Authors:** Thomas A. Dewland, Kirsten Bibbins-Domingo, Feng Lin, Eric Vittinghoff, Elyse Foster, Kofo O. Ogunyankin, Joao A. Lima, David R. Jacobs, Donglei Hu, Esteban G. Burchard, Gregory M. Marcus

**Affiliations:** 1 Electrophysiology Section, Division of Cardiology, Department of Medicine, University of California San Francisco, San Francisco, California, United States of America; 2 Knight Cardiovascular Institute, Oregon Health & Science University, Portland, Oregon, United States of America; 3 Department of Medicine, University of California San Francisco, San Francisco, California, United States of America; 4 Department of Epidemiology and Biostatistics, University of California San Francisco, San Francisco, California, United States of America; 5 Division of Cardiology, Department of Medicine, University of California San Francisco, San Francisco, California, United States of America; 6 First Cardiology Consultants, Ikoyi, Lagos, Nigeria; 7 Division of Cardiology, Department of Medicine, Johns Hopkins Hospital and School of Medicine, Baltimore, Maryland, United States of America; 8 Division of Epidemiology and Community Health, University of Minnesota, Minneapolis, Minnesota, United States of America; 9 Department of Medicine, Institute for Human Genetics, University of California San Francisco, San Francisco, California, United States of America; Cincinnati Children's Hospital Medical center, UNITED STATES

## Abstract

Whites have an increased risk of atrial fibrillation (AF) compared to Blacks. The mechanism underlying this association is unknown. Left atrial (LA) size is an important AF risk factor, and studies in older adults suggest Whites have larger LA diameters. However, because AF itself causes LA dilation, LA size differences may be due to greater subclinical AF among older Whites. We therefore assessed for racial differences in LA size among young adults at low AF risk. The Coronary Artery Risk Development in Young Adults (CARDIA) study enrolled White and Black participants between 18 and 30 years of age. LA diameter was measured in a subset of participants using echocardiography at Year 5 (n = 4,201) and Year 25 (n = 3,373) of follow up. LA volume was also assessed at Year 5 (n = 2,489). Multivariate linear regression models were used to determine the adjusted association between race and LA size. In unadjusted analyses, mean LA diameter was significantly larger among Blacks compared to Whites both at Year 5 (35.5 ± 4.8 mm versus 35.1 ± 4.5 mm, p = 0.01) and Year 25 (37.4 ± 5.1 mm versus 36.8 ± 4.9 mm, p = 0.002). After adjusting for demographics, comorbidities, and echocardiographic parameters, Whites demonstrated an increased LA diameter (0.7 mm larger at Year 5, 95% CI 0.3–1.1, p<0.001; 0.6 mm larger at Year 25, 95% CI 0.3–1.0, p<0.001). There was no significant association between race and adjusted Year 5 LA volume. In conclusion, in a young, well-characterized cohort, the larger adjusted LA diameter among White participants suggests inherent differences in atrial structure may partially explain the higher risk of AF in Whites. The incongruent associations between race, LA diameter, and LA volume suggest that LA geometry, rather than size alone, may have implications for AF risk.

## Introduction

Although atrial fibrillation (AF) is the most commonly encountered clinical arrhythmia, the factors responsible for its induction and perpetuation remain incompletely understood [1]. Compared to Whites, Blacks have more established AF risk factors yet paradoxically exhibit a 25–40% reduced risk of the disease [2,3]. Indeed, both White race and European ancestry are independently associated with AF risk [2–4]. While the strength of the race-AF association suggests the pathways that mediate this relationship play an important role in AF pathogenesis, these mechanisms remain entirely unknown.

In addition to race, increased left atrial (LA) size imparts substantial AF risk [5–7]. Previous studies have found that Whites may have significantly larger LA diameters compared to Blacks [8,9], raising the possibility that the race-AF association could be mediated by inherent racial differences in atrial size. However, prior investigations have enrolled predominantly elderly patients at heightened risk of AF. Since AF itself may also *cause* LA enlargement [10–12], it remains unclear whether these observed differences in atrial size by race are instead an artifact of a greater subclinical AF burden among older Whites compared to Blacks.

Comparison of LA size between Black and White individuals without AF could clarify whether subclinical AF contributes to atrial enlargement in these previously studied populations; the presence of LA size differences by race in a young population at low risk for AF would suggest that subclinical AF cannot explain these measured differences in atrial size. We therefore used the Coronary Artery Risk Development in Young Adults (CARDIA) study to characterize the association between race, LA diameter, and LA volume among a population-based cohort of young adults with a low burden of cardiovascular disease. To further explore this association, we also sought to determine whether percent European ancestry within Blacks is predictive of LA size. We hypothesized that LA diameter and volume measurements would be larger among Whites and increase with percent European ancestry in this young cohort at low risk for AF.

## Methods

CARDIA is a prospective, community-based cohort study sponsored by the National Heart, Lung, and Blood Institute. Eligibility, enrollment, and follow-up protocols have been previously published [13]. Briefly, 5,115 Black and White individuals 18 to 30 years of age were recruited between 1985 and 1986 from four medical centers (Birmingham, Alabama; Chicago, Illinois; Minneapolis, Minnesota; and Oakland, California). At baseline, all participants underwent a medical history, physical exam, and laboratory testing. Participants were then prospectively followed and measurements were repeated at Years 2, 5, 7, 10, 15, 20, and 25. In addition, participants underwent transthoracic echocardiography at Year 5 and Year 25.

All measurements were made according to a protocol standardized across the four enrollment centers. Race was self-reported by study participants. Resting heart rate in beats/min was assessed by measuring the pulse over 30 seconds. Blood pressure was determined by obtaining three successive measurements at one-minute intervals with the participant in a seated position; recorded values were derived from the average of the second and third measurements. Height and weight were obtained in light clothing. Participants were considered active smokers if they smoked more than 5 cigarettes per week. Alcohol consumption was derived from the participant’s self-reported intake of beer, wine, and liquor and expressed in drinks/week as previously described [14].

Genome-wide data was obtained among Black participants using a microarray platform (Genome-Wide Human SNP Array 6.0, Affymetrix, Santa Clara, CA) as part of the National Heart, Lung, and Blood Institute's Candidate Gene Association Resource (CARe) study [15]. HapMap CEU (Utah residents with northern and western European ancestry) and YRI (Yoruba ethnic group, Ibadan, Nigeria) populations served as European and African ancestral reference populations, respectively. The mean locus-specific percent European ancestry from 601,241 autosomal SNPs was calculated with LAMP-LD [16]. To determine the mean percent European ancestry for each participant, locus-specific ancestry was averaged across the entire genome.

Study participants underwent two-dimensional echocardiography at Year 5 (Acuson, Siemens Healthcare, Erlangen, Germany) and Year 25 (Artida, Toshiba Medical Systems, Otawara, Japan) [17]. Echocardiograms were analyzed in core laboratories at the University of California, Irvine (Year 5) and the Johns Hopkins University (Year 25). LA diameter was measured from the leading edge of the posterior aortic wall to the leading edge of the posterior LA wall using two-dimensional guided M-mode echocardiography in a standard parasternal long-axis view. LA volume was calculated by multiplying the M-mode LA diameter by the two-dimensional area of the LA in the apical four-chamber view by π/6 [18]. Left ventricular (LV) mass was calculated using the Devereux formula as previously described [17]. LV ejection fraction (EF) was assessed at Year 5 with the Teichholz technique using M-mode echocardiography in a parasternal window [19], while LVEF was quantified at Year 25 using two-dimensional four-chamber apical views [20].

Continuous variables with normal distributions are presented as means ± standard deviations (SD) and were compared using t tests. Non-normally distributed continuous variables are presented as medians with interquartile ranges (IQR) and were compared using Wilcoxon rank-sum tests. The association between categorical variables was determined using Chi-squared tests. Linear regression models were used to determine the association between race and LA size both before and after controlling for clinical variables likely to differ by race and known to be associated with LA size [21], including age [22], gender [23], smoking status [22], alcohol consumption [24], body mass index [23], heart rate [25], systolic blood pressure [22], antihypertensive treatment[26], ejection fraction [23], and left ventricular mass [27].

**Fig 1:**
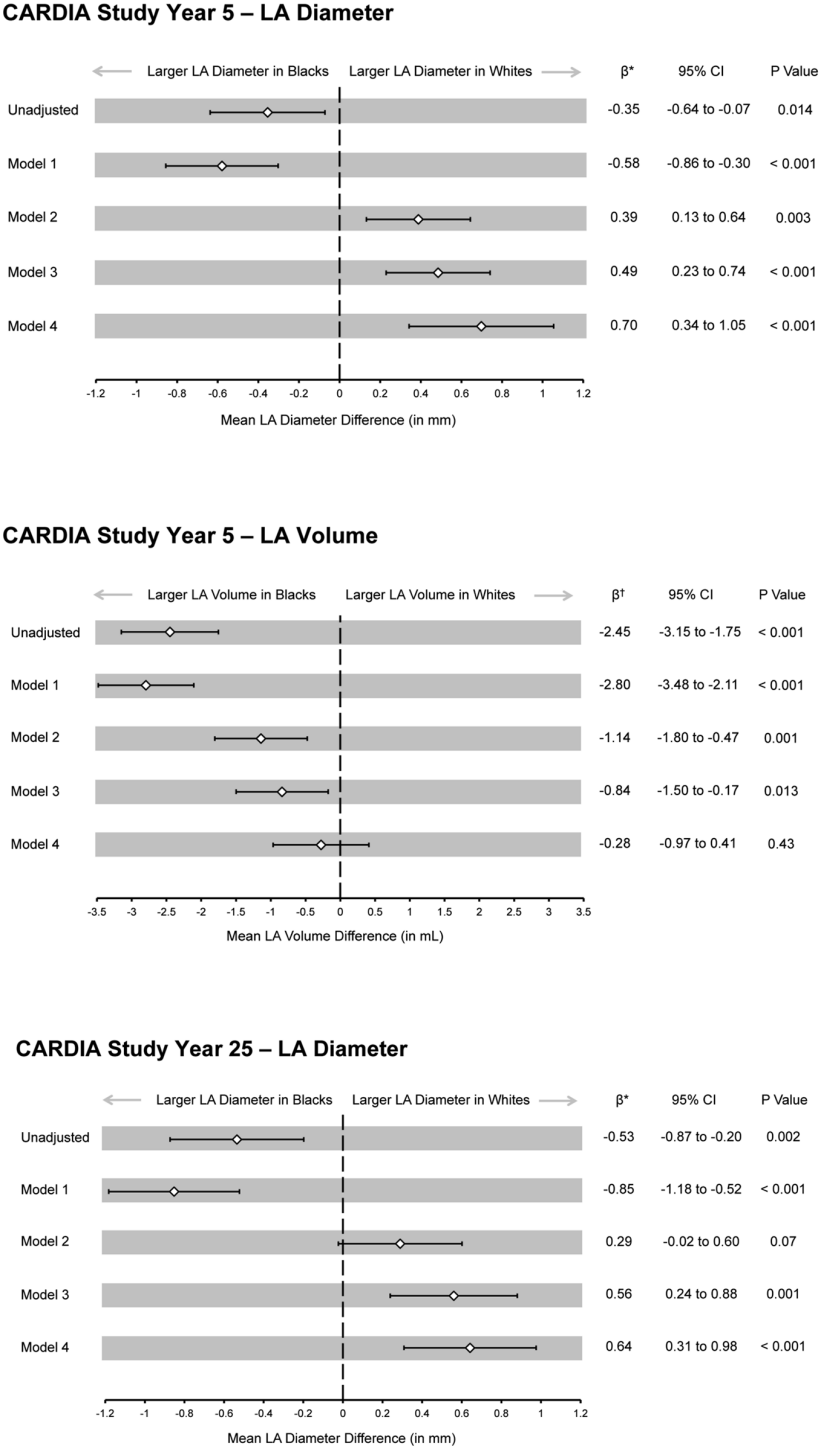
Association Between Race and Left Atrial Size. Model 1: Adjusted for age and gender. Model 2: Adjusted for Model 1 variables, smoking status, alcohol consumption, and body mass index. Model 3: Adjusted for Model 2 variables, heart rate, systolic blood pressure, and antihypertensive treatment. Model 4: Adjusted for Model 3 variables, left ventricular mass, and left ventricular ejection fraction. * Mean millimeter difference in left atrial diameter between Black and White participants. † Mean milliliter difference in left atrial volume between Black and White participants. CI, confidence interval; LA, left atrium; mL, milliliter; mm, millimeter.

Confounding variables were grouped and added to successive regression models to better understand the iterative effects of certain classes of covariates on the measured outcomes (Fig 1). Linear regression models were also used to determine the association between percent European ancestry and LA size. Because the distribution of European ancestry was right skewed, we also assessed the associations between ancestry, LA diameter, and LA volume after both log-transformation and categorization (by quartile) of this predictor.

All clinical variables were assessed at both Year 5 and Year 25. Separate analyses were performed at each study time point using covariate and echo data obtained during the same study visit. Individuals without a LA diameter measurement on the Year 5 or Year 25 echocardiogram were excluded from the diameter analysis for the respective year. Due to protocol differences, two-dimensional LA area was not measured in the apical four-chamber view on the Year 25 echocardiogram. As a result, volume analyses were limited to participants with both LA diameter and LA area measurements on the Year 5 examination only. Because clinical evidence [5] and current models of AF pathophysiology identify absolute LA diameter or volume (versus LA dimension corrected for body surface area) as the primary atrial-based determinant of AF risk, our outcome was a non-indexed LA diameter or volume.

Data were analyzed using Stata 12.0 (StataCorp, College Station, TX, USA). A two-tailed p < 0.05 was considered statistically significant. Certification to use deidentified CARDIA data was obtained from the University of California, San Francisco Committee on Human Research.

## Results

### Study Groups

Of the 4,352 participants examined at the Year 5 visit, 4,243 underwent echocardiography and 4,201 Black and White participants had adequate assessment of LA diameter. Of the 3,498 participants attending the Year 25 examination, 3,474 underwent echocardiography and 3,373 had adequate assessment of LA diameter. Mean age at the Year 5 and Year 25 visits was 30 ± 4 and 50 ± 4 years, respectively. Maximal participant age at the Year 5 and Year 25 visits was 36 and 56 years, respectively. Compared to Whites, Blacks demonstrated a significantly higher mean BMI, had an increased mean systolic blood pressure, were more likely to receive pharmacologic antihypertensive therapy, more frequently smoked cigarettes, and had a higher LV mass (Table 1).

**Table 1:** Baseline Characteristics of CARDIA Participants by Race*.

	Black (n = 2,038)	White (n = 2,163)	P value^†^
Age, years, mean ± SD	29 (4)	30 (3)	< 0.001
Male Gender, n (%)	872 (43)	1,023 (47)	0.003
Active Smoker, n (%)	699 (34)	498 (23)	< 0.001
Alcohol Consumption, drinks/week, median (IQR)	0 (0–5)	2 (0–6)	< 0.001
Body Mass Index, kg/m^2^, mean ± SD	27.4 (6.7)	24.9 (4.7)	< 0.001
Heart Rate, beats/minute, mean ± SD	69 (10)	68 (10)	0.03
Systolic Blood Pressure, mm Hg, mean ± SD	110 (12)	106 (11)	< 0.001
Antihypertensive Treatment, n (%)	52 (3)	16 (1)	< 0.001
Ejection Fraction, %, mean ± SD	63 (7)	63 (6)	0.32
Left Ventricular Mass, grams, mean ± SD	153 (46)	146 (43)	< 0.001

* Data describes the 4,201 participants who underwent the Year 5 echocardiographic examination with analyzable LA diameter data.

^†^ P values are for the comparison of the indicated characteristic in Black versus White participants.

IQR, interquartile range; SD, standard deviation.

### Baseline Characteristics and LA Diameter

In bivariate analyses, nearly all of the traditional risk factors for atrial enlargement were significantly associated with LA diameter at both Year 5 and Year 25 (Table 2).

**Table 2:** Unadjusted Association Between Traditional Risk Factors and Left Atrial Diameter.

	CARDIA Study Year 5			CARDIA Study Year 25		
Risk Factor	β*	95% CI	P value	β*	95% CI	P value
Age (per 10 years)	0.9	0.5 to 1.3	< 0.001	1.0	0.5 to 1.4	< 0.001
Male Gender	2.4	2.1 to 2.6	< 0.001	2.5	2.2 to 2.9	< 0.001
Active Smoker	0.4	0.1 to 0.7	< 0.001	0.01	-0.4 to 0.5	0.97
Alcohol Consumption (per 5 drinks/week)	0.2	0.1 to 0.2	< 0.001	0.1	-0.3 to 0.2	0.172
Body Mass Index (per 5 kg/m^2^)	1.7	1.6 to 1.8	< 0.001	3.1	2.9 to 3.4	< 0.001
Heart Rate (per 10 beats/min)	-0.6	-0.8 to -0.5	< 0.001	-0.4	-0.5 to -0.2	< 0.001
Systolic Blood Pressure (per 10 mmHg)	0.9	0.8 to 1.1	< 0.001	0.7	0.6 to 0.8	< 0.001
Antihypertensive Treatment	3.2	2.0 to 4.3	< 0.001	2.0	1.6 to 2.3	< 0.001
Ejection Fraction (per 10%)	0.3	-0.02 to 0.6	0.07	-0.4	-0.6 to -0.2	0.001
Left Ventricular Mass (per 10 grams)	0.6	0.5 to 0.6	< 0.001	0.5	0.4 to 0.5	< 0.001
Black Race	0.4	0.1 to 0.6	0.01	0.5	0.2 to 0.9	0.002

* Mean millimeter increase in left atrial diameter per unit change in baseline characteristic.

### Race and LA Diameter

In unadjusted analyses, mean LA diameter was significantly larger among Blacks compared to Whites both at Year 5 (35.5 ± 4.8 mm versus 35.1 ± 4.5 mm, p = 0.01) and Year 25 (37.4 ± 5.1 mm versus 36.8 ± 4.9 mm, p = 0.002). After adjusting for the demographic, medical comorbidity, and echocardiographic variables listed in Table 2, Whites demonstrated a significantly increased LA diameter at both Year 5 and Year 25 (Fig 1). The mean difference in LA diameter by race increased after controlling for alcohol and tobacco consumption, body mass index, hemodynamic data, and echocardiographic parameters. The same pattern was observed for the Year 25 LA diameter data. When the race-LA diameter association was adjusted only for LV mass, there was no significant difference in atrial size between races (Year 5: Whites 0.04 mm larger, 95% CI -0.21 to 0.27, p = 0.78, Year 25: Whites 0.17 mm larger, 95% CI -0.14 to 0.48, p = 0.28).

On average, LA diameter increased by 1.9 ± 5.0 mm between the Year 5 and Year 25 examinations. Year 5 LA diameter was significantly associated with Year 25 diameter; for each 1 millimeter increase in Year 5 diameter the Year 25 diameter increased by 0.4 mm (95% CI 0.4 to 0.5, p < 0.001). The unadjusted mean change in LA diameter over 20 years of follow up was not significantly different between races (1.8 ± 4.8 mm in Whites versus 2.0 ± 5.4 mm in Blacks, p = 0.25).

### Race and LA Volume

**Fig 2:**
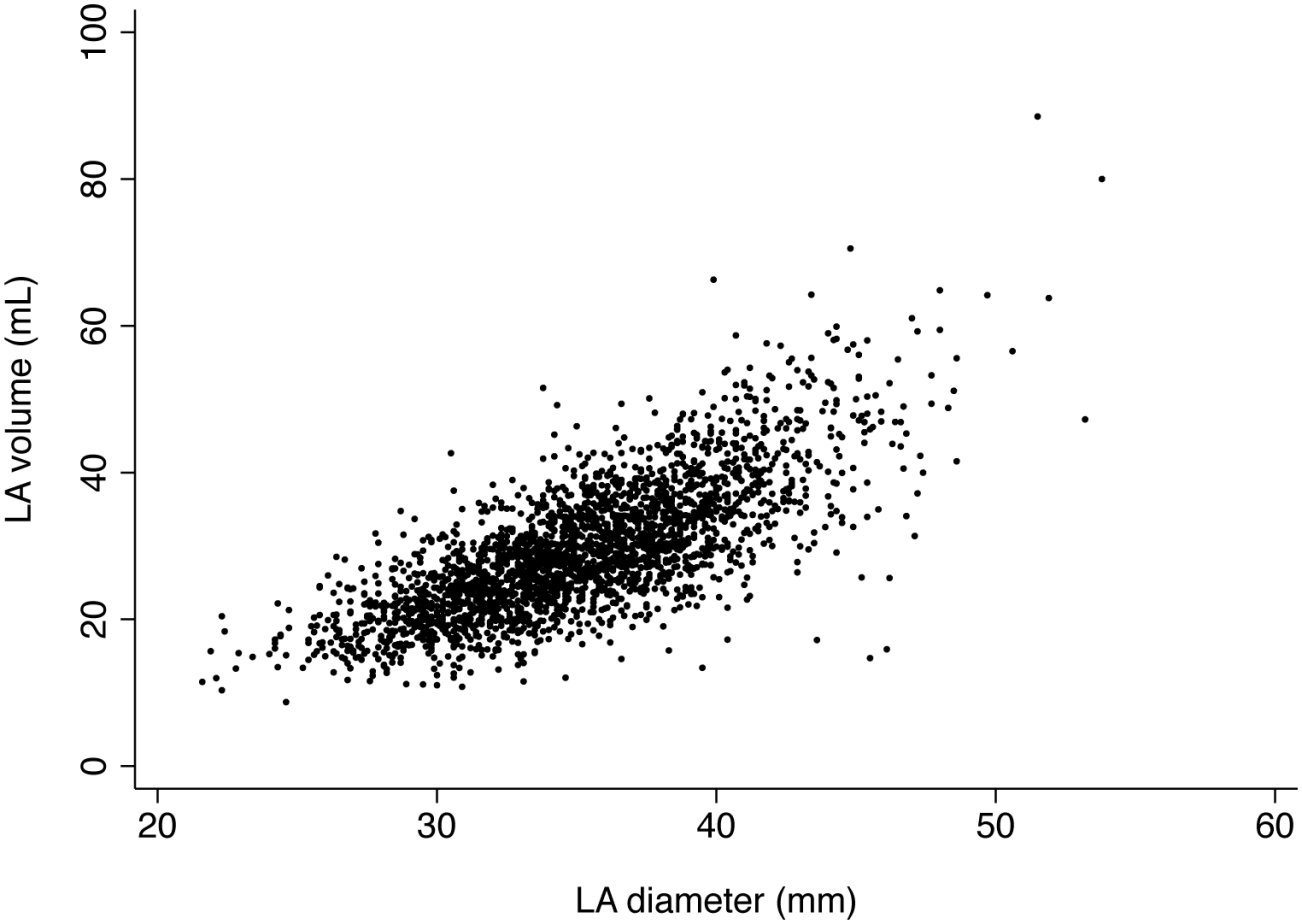
Correlation Between LA Volume and LA Diameter on the Year 5 Echocardiogram. Correlation between LA volume and LA diameter among 2,489 White and Black participants (r = 0.75, p < 0.001). Both measurements were obtained on the Year 5 echocardiogram. LA, left atrium; mL, milliliter; mm, millimeter.

A total of 2,489 participants (1,243 Black, 50%) underwent LA volume assessment on the Year 5 echocardiogram. There was a strong and significant correlation between the Year 5 LA diameter and LA volume measurements (r = 0.75, p < 0.001, Fig 2). In unadjusted analyses, LA volume was significantly larger among Blacks compared to Whites (30.7 ± 9.5 mL versus 28.2 ± 8.2 mL, p < 0.001). After controlling for LV mass alone, mean LA volume remained, on average, 1.4 mL larger in Blacks (95% CI 0.9 to 2.0 mL, p < 0.001). With full adjustment for the clinical and echocardiographic variables in Table 2, the larger mean LA volume among Blacks was no longer statistically significant (Fig 1).

### European Ancestry and LA Size

European ancestry data was available for 767 of the 2,038 Black participants (38%) with available Year 5 LA diameter data, 669 of the 1,580 Black participants (42%) with Year 25 LA diameter data, and 443 of the 1,243 Black participants (36%) with Year 5 LA volume data. Median percent European ancestry among Black participants was 17% (IQR 12–24%). We did not observe a significant association between percent European ancestry and LA diameter at either study time point; at Year 5, each 10 percent absolute increase in European ancestry was associated with a non-significant 0.1 mm decrease in LA diameter (-0.10, 95% CI -0.38 to 0.18, p = 0.50), while at Year 25 each 10 percent absolute increase in European ancestry was associated with a non-significant 0.02 mm increase in LA diameter (0.02, 95% CI -0.32 to 0.37, p = 0.90). Similarly, there was no significant association between European ancestry and LA volume; each 10 percent absolute increase in European ancestry was associated with a 0.08 mL decrease in LA volume (-0.08, 95% CI -0.75 to 0.59, p = 0.82). These associations were not meaningfully changed after adjustment for the variables listed in Table 2 or when percent European ancestry estimates were either log-transformed or categorized by quartile (S1 Table).

## Discussion

In a large, population-based cohort of young adults, we observed a larger unadjusted LA diameter among Black versus White participants. After controlling for several demographic variables and clinical comorbidities, however, adjusted LA diameter was significantly greater among Whites. Although racial differences in atrial diameter were consistent across two serial echocardiographic assessments separated by 20 years, they were notably modest in magnitude. Furthermore, we did not identify a significant association between race and adjusted LA volume, nor was there a discernable relationship between percent European ancestry and LA size (diameter or volume) within Black participants.

Differences in LA diameter between races have been observed in cohorts of older adults (mean age > 65 years) with a high burden of cardiovascular disease. Data from both the Cardiovascular Health Study [8] and the Heart and Soul Study [9] indicate that older Whites exhibit an approximate 2 mm larger left atrial diameter compared to Blacks. As a 5 mm larger LA diameter has been associated with a 40% heightened AF risk [5], these prior findings might explain the substantially elevated risk of AF among Whites compared to non-Whites. AF itself, however, is a known cause of LA dilation, and arrhythmia detection can be difficult when episodes are paroxysmal and asymptomatic. Therefore, in these previously studied populations at high risk for the development of AF, it is unclear if subclinical disease can explain the observed racial differences in atrial diameter.

In the present investigation, we observed a statistically significant larger adjusted LA diameter among Whites on both the Year 5 and Year 25 exams. It is notable that the absolute adjusted difference in LA diameter between races was similar on both the Year 5 and Year 25 exams, suggesting that racial differences in LA size are present at a young age and remain stable over time. Recent evidence indicates that racial differences in AF risk diminish in the setting of comorbid diseases [4], suggesting that race may play an especially important role in driving risk among patients with lone AF. These LA diameter results support this observation, as differences in atrial diameter between races were largest after controlling for a variety of pathologic factors known to increase LA diameter and diminished or disappeared in the presence of AF risk factors. Furthermore, the present study provides compelling evidence that previously described racial differences in LA diameter [8,9] are not entirely explained by poor AF ascertainment. However, it should be recognized that the absolute difference in mean adjusted atrial diameter at each time point (0.70 mm larger in Whites at Year 5, 0.64 mm at Year 25) in the current investigation was smaller than previously reported in cohorts of older patients [8,9]. In light of prior data relating absolute atrial size and AF risk, the differences in diameter observed in the present study are unlikely to fully explain the approximate 25–40% reduction in AF risk previously described among Blacks compared to Whites [2,3]. While it remains possible that atrial size disparities by race become more evident and clinically relevant with advanced age, it is clear that there is not a large racial difference in LA diameter among young adults.

Contrary to previous investigations, Black participants in our study had a significantly larger *unadjusted* LA diameter on both the Year 5 and Year 25 echocardiograms. This finding could be attributed to racial and age-related differences in LV mass. Ventricular hypertrophy, which is quantified by measurement of LV mass, influences LA size via its contribution to diastolic dysfunction. Blacks had a significantly increased LV mass on both the Year 5 and Year 25 echocardiograms, and after adjustment for this single variable there was no longer a significant difference in LA diameter by race at either time point. It is likely that LV mass represents a readily quantifiable “downstream” measurement that is effected by a variety of pathologic pathways, including hypertension. Notably, prior studies among *older* populations have not identified significant differences in LV mass by race [9,28]. It is therefore possible that racial differences in LV mass among younger individuals explains the larger unadjusted mean atrial diameter among Blacks in our study, while equalization of ventricular mass at advanced age accounts for the unadjusted findings from previous investigations performed in older patients. Alternatively, increased mortality among younger Blacks with left ventricular hypertrophy [29] (and LA enlargement) could account for differences in LV mass and unadjusted atrial size between the CARDIA cohort and these more elderly study populations.

In addition to atrial diameter, we also measured LA volume on the Year 5 echocardiogram. While prior investigations have established LA diameter as a significant, independent risk factor for incident AF [5,6], more recent data suggests LA volume may be superior for predicting a composite cardiovascular endpoint of AF, heart failure, stroke, transient ischemic attack, myocardial infarction, coronary revascularization, or cardiovascular death [25]. In the present study we did not identify a significant difference in adjusted LA volume between young White and Black participants on the Year 5 exam, and the 95% confidence intervals surrounding this estimate exclude a large, clinically meaningful difference in LA volume between races in early adulthood. It is presently unclear why the comparison of adjusted LA diameter and volume between races did not yield qualitatively consistent results, although race-specific differences in LA geometry could be postulated. Because LA volume requires two separate echocardiographic measurements for its calculation (LA diameter in the parasternal short axis view and LA area in the apical four-chamber view), this parameter may be more prone to bias than LA diameter, especially in obese or otherwise difficult to image patients. Although LA diameter was previously found to be significantly larger among Whites versus Blacks enrolled in the Heart and Soul Study, there was similarly no observed difference in LA volume between races [9]. As LA diameter has been reproducibly associated with incident AF [5,6], these findings suggest that diameter may specifically capture an important aspect of atrial geometry that mediates racial differences in AF risk. In addition, this could imply that LA morphology, rather than volume (or size) alone, is particularly important in imparting AF risk for all susceptible patients.

When our analysis was restricted to Black participants, we did not observe a significant association between percent European ancestry and either of the LA size measurements. The confidence intervals surrounding these point estimates suggest a clinically important association between European ancestry and LA size is very unlikely. We notably used a two-population model to calculate European ancestry; in this setting, European ancestry and African ancestry are perfectly inversely correlated. The association between African ancestry and atrial size among Blacks would therefore yield identical results (but with opposite beta coefficients). In light of prior data linking relatively small increases in European ancestry to AF risk, these results lend further support to the notion that atrial size differences do not adequately explain the substantially heightened AF risk among Whites. Since only a modest proportion of Blacks had percent European ancestry calculations, the resultant loss of power may have diminished our ability to identify significant relationships (i.e. type II error). Alternatively, racial differences in LA diameter may be primarily determined by environmental exposures that are not proportional to genetic ancestry.

Potential limitations of our analysis should be recognized. We sought to characterize racial differences in LA size in a young cohort with a low prevalence of cardiovascular comorbidities to eliminate the potential bias introduced by subclinical AF. However, AF has not been systematically ascertained in the CARDIA cohort and it was not possible to identify the likely small number of patients with this diagnosis. Because the prevalence of AF among individuals < 55 years old is exceedingly low (0.1% for women, 0.2% for men) [30] and the oldest participants for the Year 5 and Year 25 echocardiograms were 36 and 56 years old, respectively, we believe it is unlikely that this potential source of bias could substantially impact our results. Due to slight differences in echocardiogram protocols between study visits, LA volume could only be calculated on the Year 5 examination. As noted above, it remains possible that differences in LA volume by race may become more manifest in older adults. We did not control for diastolic function in our multivariate models, although the prevalence of this abnormality in the CARDIA cohort is low and LV mass may have provided a sufficient surrogate [18]. Finally, race was self-reported by the study participants. It should be noted that the vast majority of studies examining racial differences in AF have used this methodology and that inaccurate self-reporting of race would likely bias our results towards the null. In addition, our ancestry data confirmed that the majority of self-identified Black participants were of predominately African descent.

In a large, multicenter, population-based cohort study of young adults, we observed a larger unadjusted LA diameter among Black versus White participants. After adjusting for clinical comorbidities, however, adjusted LA diameter was significantly greater among Whites and this difference persisted over a 20-year follow up period. A similar adjusted difference in LA volume by race, however, was not observed. Although atrial diameter may partially mediate the association between race and AF, the magnitude of this atrial size difference likely does not adequately explain the substantially heightened AF risk among Whites. Incongruent LA diameter and volume findings by race validate previous findings and suggest that LA geometry, rather than size alone, may have important implications for AF risk.

## Supporting Information

S1 Table Association Between European Ancestry and Left Atrial Size. (DOCX)
