# Supplementary material for: Racial Differences in Left Atrial Size: Results from the Coronary Artery Risk Development in Young Adults (CARDIA) Study
Source: PLoS One. 2016 Mar 17;11(3):e0151559. doi: 10.1371/journal.pone.0151559 (PMC4795666; doi:10.1371/journal.pone.0151559)
Supplement: S1 Table — (DOCX) [file pone.0151559.s001.docx]

**S1 Table.** Association Between European Ancestry and Left Atrial Size

|  | **CARDIA Study Year 5** | | | | | | |  | **CARDIA Study Year 25** | | |
| --- | --- | --- | --- | --- | --- | --- | --- | --- | --- | --- | --- |
|  | LA Diameter | | |  | LA Volume | | |  | LA Diameter | | |
| **European Ancestry** | β* | 95% CI | P value |  | β^†^ | 95% CI | P value |  | β* | 95% CI | P value |
|  |  |  |  |  |  |  |  |  |  |  |  |
|  |  |  |  |  |  |  |  |  |  |  |  |
| Unadjusted Association | -0.10 | -0.38 to 0.18 | 0.50 |  | -0.08 | -0.75 to 0.59 | 0.82 |  | 0.02 | -0.32 to 0.37 | 0.90 |
| Adjusted Association^‡^ | -0.01 | -0.35 to 0.32 | 0.93 |  | -0.06 | -0.66 to 0.55 | 0.86 |  | -0.06 | -0.38 to 0.26 | 0.71 |
| Adjusted Log-Transformed Association^‡^ | 0.07 | -0.61 to 0.74 | 0.85 |  | -0.31 | -1.56 to 0.93 | 0.63 |  | -0.09 | -0.74 to 0.56 | 0.78 |
| Adjusted Categorical Association^‡^ |  |  |  |  |  |  |  |  |  |  |  |
| Quartile 1 | Reference | | |  | Reference | | |  | Reference | | |
| Quartile 2 | 0.29 | -0.78 to 1.36 | 0.59 |  | 0.15 | -1.82 to 2.13 | 0.88 |  | 0.03 | -0.99 to 1.05 | 0.96 |
| Quartile 3 | 1.09 | -0.01 to 2.19 | 0.05 |  | 0.58 | -1.45 to 2.61 | 0.57 |  | 0.23 | -0.78 to 1.23 | 0.66 |
| Quartile 4 | -0.24 | -1.32 to 0.83 | 0.66 |  | -0.02 | -2.02 to 1.97 | 0.98 |  | -0.19 | -1.20 to 0.82 | 0.71 |

In models with a continuous European Ancestry measurement, the resultant beta coefficient represents the change in atrial size per each 10% absolute increase in percent European ancestry.

*Mean millimeter increase in left atrial diameter.

†Mean milliliter increase in left atrial volume.

‡Adjusted for age, gender, smoking status, alcohol consumption, body mass index, heart rate, systolic blood pressure, antihypertensive treatment, ejection fraction, and left ventricular mass.
